# Assessing initial plan check efficacy using TG 275 failure modes and incident reporting

**DOI:** 10.1002/acm2.13640

**Published:** 2022-05-10

**Authors:** Adam C. Riegel, Cynthia Polvorosa, Anurag Sharma, Jameson Baker, William Ge, Joseph Lauritano, Emel Calugaru, Jenghwa Chang, Jeffrey Antone, Angela Oliveira, Walkiria Buckenberger, William Chen, Yijian Cao, Ajay Kapur, Louis Potters

**Affiliations:** ^1^ Department of Radiation Medicine Northwell Health Lake Success New York USA; ^2^ Donald and Barbara Zucker School of Medicine at Hofstra/Northwell Hempstead New York USA

**Keywords:** Chart checking, incident reporting, patient safety, quality management

## Abstract

Plan checks are important components of a robust quality assurance (QA) program. Recently, the American Association of Physicists in Medicine (AAPM) published two reports concerning plan and chart checking, Task Group (TG) 275 and Medical Physics Practice Guideline (MPPG) 11.A. The purpose of the current study was to crosswalk initial plan check failure modes revealed in TG 275 against our institutional QA program and local incident reporting data. Ten physicists reviewed 46 high‐risk failure modes reported in Table S1.A.i of the TG 275 report. The committee identified steps in our planning process which sufficiently checked each failure mode. Failure modes that were not covered were noted for follow‐up. A multidisciplinary committee reviewed the narratives of 1599 locally‐reported incidents in our Radiation Oncology Incident Learning System (ROILS) database and categorized each into the high‐risk TG 275 failure modes. We found that over half of the 46 high‐risk failure modes, six of which were top‐ten failure modes, were covered in part by daily contouring peer‐review rounds, upstream of the traditional initial plan check. Five failure modes were not adequately covered, three of which concerned pregnancy, pacemakers, and prior dose. Of the 1599 incidents analyzed, 710 were germane to the initial plan check, 23.4% of which concerned missing pregnancy attestations. Most, however, were caught prior to CT simulation (98.8%). Physics review and initial plan check were the least efficacious checks, with error detection rates of 31.8% and 31.3%, respectively, for some failure modes. Our QA process that includes daily contouring rounds resulted in increased upstream error detection. This work has led to several initiatives in the department, including increased automation and enhancement of several policies and procedures. With TG 275 and MPPG 11.A as a guide, we strongly recommend that departments consider an internal chart checking policy and procedure review.

## 1 INTRODUCTION

Planning and delivering radiation therapy is a complex process that involves numerous people filling different roles on a patient care team. Ensuring high‐quality care requires a rigorous quality management program to help mitigate the risk of harm. Designing a quality management program can be a daunting task, particularly deciding where limited resources should be allocated in mitigating risk. The American Association of Physicists in Medicine (AAPM) has advocated the failure modes and effects analysis (FMEA) methodology^1^ to identify areas of significant risk so institutions may allocate resources to efficiently reduce it. Recently, the AAPM published a new TG report that utilized FMEA to generate recommendations for physics plan and chart review.^2^ In addition to reporting high‐risk failure modes and causes learned during its FMEA, TG 275 also surveyed clinical medical physicists regarding their current chart check practices, compared failure modes against national and international error reporting databases, and recommended key practices that could improve physics chart review. Closely related is the recently‐published Medical Physics Practice Guideline (MPPG) which provides further recommendations on how the findings of TG 275 can be implemented clinically.^3^

An important aspect of quality management is periodic review of the program's policies and procedures to ensure continued improvement in the delivery of high‐quality care at minimal risk despite inevitable departmental changes over time.^1^ Over the past decade, our institution has applied FMEA to the radiation oncology process.^4^, ^5^ These analyses have resulted in significant changes in process and culture in our department, including the development of the “No‐Fly” process,^6^ the standardization of treatment pathways,^7^ and the implementation of daily prospective contour rounds.^8^, ^9^, ^10^ TG 275 presented a timely opportunity to review our policies and procedures with the benefit of collected experience external to our department and consensus recommendations.

The TG report recommended that individual departments tailor recommendations of the report to specific risks encountered locally and review reported incidents within the department to ensure efficacy of the quality program.^2^ The purpose of the current work is to report our initial experience in applying the findings of TG 275 to the external beam radiation therapy initial plan check. The work is divided into two parts: first, a cross‐comparison of high‐risk failure modes and causes against our current radiation oncology workflow to determine opportunities for improvement in our plan check process, and second, a comparison of locally reported incidents to validate our cross‐comparison.

## 2 METHODS

### 2.1 Clinical workflow

**FIGURE 1:**
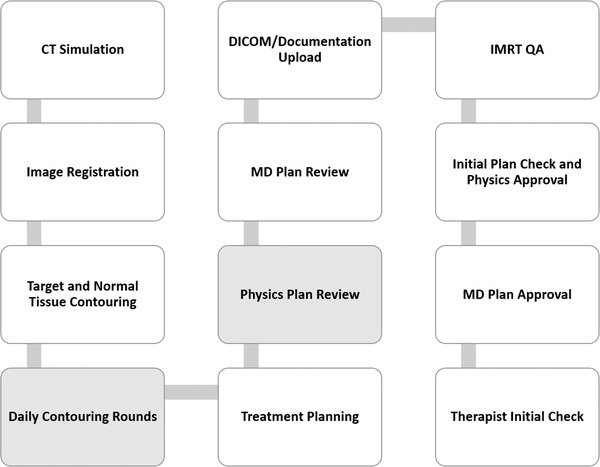
Treatment planning workflow at our institution. Shaded boxes highlight additional quality assurance steps compared with the conventional treatment planning process

As recognized in TG 275, the clinical workflow can vary significantly from department to department.^2^ The clinical treatment planning workflow in our department is diagrammatically represented in Figure 1. Two steps in our process that differ from other departments are Daily Contouring Rounds and Physics Plan Review. Both are described below.

Prior to 2012, our department had weekly new‐start chart rounds. We found, however, that issues identified at chart rounds rarely resulted in a change of care since treatment had already begun. In 2012, we instituted daily contouring rounds with peer review upstream in the planning process as a means of identifying issues prior to the start of treatment planning. Daily peer review consists of treatment prescription and contour reviews by the attending physicians, residents, physicists, and dosimetrists from all geographic locations within the department. Over time, we identified that 28% of cases required some degree of modification prior to treatment planning.^9^

Another component of our program consists of “Physics Plan Review.” Prior to having the physician review the plan, an additional review by a physicist takes place to approve the validity and veracity of the plan. Previously, our workflow had the physician review the plan, the dosimetrist would then upload the plan, and then physics would review and approve. This resulted in numerous plans being sent back to planning for technical issues discovered by the physicist, thereby incurring an additional review by the physician after the plan was modified. The purpose of the additional physics was to catch technical issues prior to physician review that may cause additional physician reviews and delays in the planning process. Both the Physics Plan Review and Initial Plan Check/Physics Approval are performed by qualified medical physicists as per MPPG 11.A recommendations.^11^

### 2.2 Analysis

In FMEA, relative risk of a failure mode and cause pair is determined by a multi‐disciplinary group quantitatively evaluating the severity, frequency of occurrence, and likelihood of a failure mode to go undetected. Group members assign numerical values to these parameters and the risk priority number is the product of these values, where higher numbers indicate greater risk.^1^

A committee of 10 physicists was established for the TG 275 review. The committee was asked to review the 46 high‐risk failure modes (risk priority numbers greater than 100) reported in Table S1.A.i of the TG report. Our local committee identified steps in our planning process which members believed sufficiently checked the failure modes presented in the TG 275 report. It should be noted that some steps in Figure 1 are singularly designed as quality assurance (QA) checkpoints such as “Initial Plan check.” Other steps are procedural in nature where the primary goal is not QA but rather to generate an important part of the treatment plan. Even procedural steps such as “Image Registration” and “Treatment Planning,” however, create an opportunity for QA by a dosimetrist or physicist who is completing the task. These procedural steps, then, were considered “checks” in the planning process which may be redundant with items in the initial physics plan check. Multiple check points could be associated with a single failure mode, but a “primary” check was defined as the most upstream check most likely to catch the error. Failure modes that were not covered by our current process were considered “gaps” and noted for follow‐up.

Our institution uses the Radiation Oncology Incident Learning System (ROILS)^12^ to record and report events with institution‐specific tags that allow us to track incident frequency over time. A subcommittee of our larger quality committee consisting of one physicist, one dosimetrist, and two radiation therapists reviewed the narratives of 1599 incidents reported from September 2019 to August 2021 for the purposes of this investigation. The subcommittee categorized each incident into the high‐risk failure modes reported in TG 275. For a subset of the most frequent failure modes that occurred in our ROILS data, the committee assessed if the event was caught before or after the primary check defined in the initial review of our QA program.

## 3 RESULTS

**FIGURE 2:**
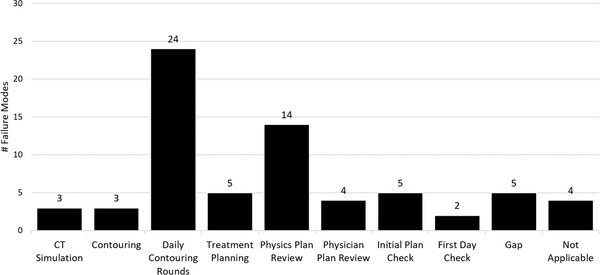
Failure modes discoverable by various steps of the treatment planning process as per an in‐house committee consisting of 10 physicists. Each failure mode could be covered by multiple quality assurance steps

Figure 2 illustrates which steps of the planning process covered the failure modes presented in Table S1.A.i in TG 275. Of the 46 high‐risk failure modes presented, five (5) were found to be inadequately covered by the quality program embedded in the treatment planning workflow (10.8%). The five failure modes and causes (reproduced from TG 275) are listed in Table 1.

**TABLE 1:** Failure modes^2^ identified as gaps in QA program

Failure Mode	Description	Cause
2	Miscommunication about prior dose, pacemaker, pregnancy	Information not communicated or available information incorrect
4	Unintentional re‐irradiation of a previously treated area	Technical issue: Inadequate medical records in hospital database, recreation of prior plan incorrect, missing previous RT dose structure, no records available (foreign country, distant past, lost)
8	Sub‐optimal treatment plan or approach related to communication or coordination with multidisciplinary care	Lack of coordination or miscommunication with, e.g., surgeons, med onc, etc.
16	Plan reviewed incorrectly by attending MD	Covering MD (not familiar with case details), MD rushed
46	Pacemaker/defibrillator patient not monitored adequately during treatment	Monitoring not requested and/or presence of device not communicated

Daily Contouring Rounds were found to be very effective in detecting initial plan check failure modes. Of the 46 failure modes, 24 (52.2%) were covered at least in part by Daily Contouring Rounds (Figure 2). Over half of these (14/24, 58.3%) were solely covered by Daily Contouring Rounds and 6 of the top 10 riskiest failure modes could be detected at this step. Top‐ten high‐risk failure modes covered by Daily Contouring Rounds included “FM1: Wrong or inaccurate MD contours,” “FM 3: Improper margins for PTV,” “FM 5: Incorrect or missing pathology,” “FM 6: Dose in plan does not match intended” (with the cause stemming from incorrect prescription provided to planner), “FM 7: Wrong or inaccurate dosimetrist contours,” and “FM 9: Plan does not reflect intent” (with the cause stemming from incomplete or incorrect planning note or prescription). Physics Plan Review accounted for 14 of 46 failure modes (30.4%), the next most comprehensive QA process, though it was only identified as the primary QA step for 7/14 failure modes. Four failure modes were determined to be inapplicable to our treatment workflow (6.5%).

**FIGURE 3:**
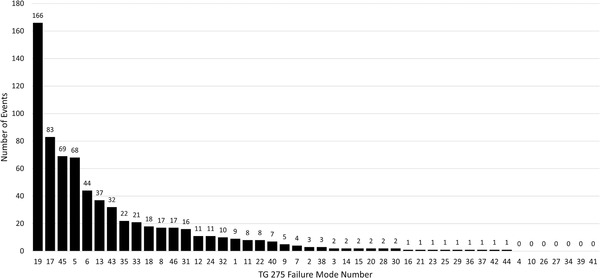
Number of events per failure mode (as numbered by AAPM TG 275) reported from September 2019 to August 2021 in our local Radiation Oncology Incident Learning System database. Classifications of event to failure mode were performed by a subcommittee of our in‐house quality assurance committee

In our analysis of 2 years of ROILS entries, over half (889) were beyond the scope of the initial plan check and therefore deemed “not applicable” for this analysis. The distribution of the remaining 710 failure modes is shown in Figure 3 in order of decreasing prevalence in the sample.

Approximately 85% of the 710 relevant ROILS entries could be associated with 13 failure modes. These are described in greater detail in Table 2. The remaining 15% had fewer than 15 entries per category over the 2‐year sample. Failure modes identified as “gaps” in the initial analysis of our QA process were not correlated with events reported in ROILS. Only two gap failure modes yielded more than 1% of the total number of events (“FM 8: Sub‐optimal treatment plan…” and “FM 46: Pacemaker/defibrillator…” at 17 events each) and the remaining three failure modes only yielded a total of four events. The most frequently reported failure mode was “FM 19: Pregnancy status not assessed” representing over 20% of the relevant entries, however 164/166 (98.8%) were detected at CT Simulation. Daily Contouring Rounds were fairly effective at identifying errors with the percentage of events reported after rounds ranging from 50% (“FM 7: Missing MD or dosimetry contours”) to 91.3% (“FM 6: Dose in plan does not match intended”). Physics Review and the traditional Initial Plan Check were less effective. For reported events where Physics Review was the primary QA step, 31.8% of reported events were detected by this check. For reported events where Initial Plan Check was the primary QA step, between 31.3%–87.5% of reported events were detected by this check. For both checks, though, the absolute number of incidents was relatively low (maximum of 91 incidents, less than 12.7% of the relevant sample).

**TABLE 2:** Most frequently reported failure modes via ROILS reporting from September 2019 to August 2021

FM #	Failure mode	Number of events	Primary QA step	Number (%) errors detected by primary QA step
19	Pregnancy status not assessed	166	CT Sim	164 (98.8%)
17	Wrong target dose	83	Daily contour rounds	61 (73.5%)
45	Miscommunication on treatment strategy from the physician to the rest of the team	69	Daily contour rounds	63 (91.3%)
5	Incorrect or missing pathology	68	CT sim	65 (95.6%)
6	Dose in plan does not match intended	44	Daily contour rounds	34 (77.3%)
13	Wrong preliminary prescription (e.g., wrong energy, dose/# fx, bolus, type of image guidance)	37	Daily contour rounds	30 (81.1%)
43	Incorrect field parameters	32	Initial plan check	10 (31.3%)
35	Suboptimal plan	22	Physics review	7 (31.8%)
33	Treatment devices omitted (such as bolus)	21	Initial plan check	12 (57.1%)
18	Missing MD or dosimetry contours	18	Daily contour rounds	9 (50.0%)
8	Sub‐optimal treatment plan or approach related to communication or coordination with multidisciplinary care	17	None*	NA*
46	Pacemaker/defibrillator patient not monitored adequately during treatment	17	None*	NA*
31	Incorrect laterality	16	Initial plan check	14 (87.5)

* These failure modes were identified as “gaps” in our QA program.

## 4 DISCUSSION

The AAPM TG 275 has identified high‐risk failure modes associated with the initial plan check and suggested specific checklist items that can mitigate risk from these failure modes.^2^ The purpose of this work was to compare failure modes determined by TG 275 against our existing treatment planning and QA process to uncover any gaps in our QA program. We found that five failure modes, three of which (FMs 2, 4, and 8) were ranked with top‐ten risk priority numbers in the TG 275 FMEA, were not adequately covered by our QA process and represent a significant opportunity for risk mitigation. Additionally, we reviewed 2 years of internal ROILS data to determine how reported events correlated with gaps in our QA program.

MPPG 11.A recommends physicists design workflows that catch errors as early as possible in the planning process.^3^ The most significant finding of our review is the efficacy of Daily Contouring Rounds in identifying errors before the treatment plan is developed. In a recent blinded study, Talcott et al. demonstrated that traditional weekly chart rounds is only 55% effective at identifying simulated errors in presented treatment plans.^13^ In response, Chera, Marks, and Potters suggest that prospective daily rounds are a potential solution, particularly in light of the technological shifts towards virtual meetings after the recent COVID‐19 pandemic.^14^ We found that Daily Contouring Rounds could detect errors associated with several high‐risk failure modes including “FM1: Wrong or inaccurate MD contours,” “FM3: Improper margins for PTV,” and “FM 7: Wrong or inaccurate dosimetrist contours.” The incidence of these events in ROILS was very low, less than 15 events in nearly 1600 entries, not because inaccurate contours did not occur, but most likely because they were constructively addressed in Daily Contouring Rounds and not reported as an “event.” Prescription failure modes (6, 13, and 17) were prevalent in the ROILS data, but most of these entries were caught in Daily Contouring Rounds (91.3%, 81.1%, and 73.5%, respectively) once again demonstrating its efficacy in catching errors upstream. We estimate that the implementation of our No‐Fly policy of prescription and contours peer review prior to planning and the insertion of the Physics Plan Review has collectively mitigated risk for 67% of the TG‐275 modes.

Though creating a physics review earlier in the process is a TG 275 and MPPG 11.A. recommendation that we have practiced for years, and, theoretically, physics review should catch a substantial number of TG 275 failure modes, our ROILS data demonstrate that the check could be more efficacious as a primary QA check. Of the top ROILS events where Physics Review was the primary QA step, only 31.8% were caught by the review. Gopan et al. studied the effectiveness of physics plan review in detecting errors and found that physics plan review detected 38% of events that could have been detected by the check, a rate similar to the findings of this study.^15^ Automation is one way to improve the detection rate.^2^ Berry et al. have shown significant improvements in error detection and efficiency using an automated plan checking tool.^16^ Xu et al. were able to automate substantial portions of the TG 275 checklist recommendations, thereby limiting manual checking effort and reducing plan check time from 44% to 98%.^17^ To increase the likelihood of error detection and reduce variability among physicists, we have designed and implemented an in‐house automated plan check script in the treatment planning system which includes many checks from the sample checklist in Table S1.A.iii in TG 275^2^ and Table 8 in MPPG 11.A.^3^ The script is designed to check several parameters manually entered in the treatment planning system against data available in the record and verify system including total dose, fraction size, the number of fractions, inclusion of correct support structure, treatment machine, and several others. The script is written in C# and takes advantage of the Eclipse Scripting Application Programming Interface (ESAPI) to run as a plugin module to Eclipse (Varian Medical Systems, Palo Alto, CA). Additionally, we are considering commercial plan/chart checking automation software for the Initial Plan Check.^18^ It should be noted that many of the errors that were missed in the Physics review and Initial Plan Check were caught by the therapist initial check (colloquially called the “pre‐flight” check), which reinforces the recommendation from MPPG 11.A that therapists perform a check prior to start of treatment.^3^ More generally, our findings support the need for redundancy in our quality management program.

Three of the five failure modes we identified as “gaps” in our QA program (FMs 2, 4, and 46) are related to what we have termed the “Three P's”: pacemaker, pregnancy, and prior treatment. In our analysis of ROILS data, we did not see many entries related to prior treatment despite lacking a well‐defined QA process when integrating prior dose. This may be due to its relatively low occurrence in the clinic or the case‐by‐case nature of incorporating prior dose warranting extra scrutiny that reduces the chance of failure. Regardless of the low number of reported incidents, we are strengthening our formal policies and procedures for incorporating prior treatment information into current treatment plans as a result of this study. In particular, we are focusing on improving written communication between physician, dosimetrists, and physicians to ensure that staff is adequately and efficiently alerted to prior treatment.

Like prior treatment, pacemaker monitoring and dosimetry are relatively infrequent requests and are often considered on a case‐by‐case basis following prior AAPM guidance.^19^ There were, however, 17 events reported regarding pacemaker dosimetry in our sample. Upon further review, we decided to update and revise our in vivo dosimetry policies and procedures for pacemaker monitoring and special measurements in general based on recent AAPM guidance.^20^, ^21^ As with prior treatment, we considered improvement in communication essential in reducing the risk of overlooking pacemaker dosimetry.

Pregnancy‐related events represented the largest fraction of ROILS entries, though not specifically FM 2 as we assessed in our initial analysis. Instead, we saw many of “FM 19: Patient pregnancy status not assessed,” where the attestation form was not completed prior to CT simulation as per our departmental policy. The overwhelming majority of these reported incidents, however, were caught at or before CT simulation (98.8%). In this case, a high reporting rate is evidence of our QA process working correctly and preventing potentially pregnant patients from CT exposure. After further consideration, we decided that this item was adequately checked by our process and did not need further action.

A drawback of the current work was the lack of local FMEA for comparison with the published consensus FMEA in TG 275. Though performing a full FMEA locally is preferred, the aim of this work was to use the failure modes published in TG 275 as a starting point for a more in‐depth review. Using the TG 275 allowed us to identify gaps in our quality management program and design policies and procedures to mend these gaps. We hope to expand our review to include a local FMEA of initial plan check, weekly check, and end of treatment check to identify failure modes that may be unique for our clinic and for comparison with TG 275.

Another weakness of the current study, indeed, of any study that utilizes incident reporting data is the variability in reporting. In our sample, most incidents were reported by radiation therapists (62.2%) which suggests a higher incidence of downstream errors appearing in the data due to their involvement later in the process. Errors more likely to occur upstream in the process may be underreported. Dosimetrists (22.3%) are the next most frequent reporter, most likely due to their role in running Daily Contouring Rounds and reporting discrepancies that occur. In addition to role‐based variation, there may be variation among individuals about what is considered an “event.” At our institution, we encourage reporting at all stages of the radiation therapy process to improve efficiency as well as safety, but there is some ambiguity about what rises to the level of reportable. Many mistakes are simply corrected and not reported, so our data may be favoring errors caught by groups with greater tendencies to report or specific types of errors that are actively monitored in the department. Ford and Evans discuss this issue, suggesting that incidents that reach the patient and “near misses” should certainly be reported, but acknowledge that personal judgment and bias are prominent factors in what reaches the database.^22^ We hope to study the reliability of self‐reported incident data in subsequent investigations.

## 5 CONCLUSION

Initial plan checks are a vital component of radiation therapy QA. Using failure modes identified in AAPM TG 275, we identified the strengths and weaknesses of our No‐Fly treatment planning QA program. Five failure modes were identified as vulnerable aspects of our QA program and we are using our findings to design QA practices to guard against these risks. We found that several initial plan check failure modes were adequately covered upstream of the conventional initial plan check, specifically noting the efficacy of Daily Contouring Rounds in identifying errors earlier in the planning process. The TG 275 assessment has led to several initiatives in the department, including increased automation, enhancement of several policies and procedures, and the undertaking of a local FMEA for initial plan check. We strongly recommend all radiation oncology departments consider an internal review of chart checking policies and procedures using TG 275 and MPPG 11.A for guidance. Furthermore, we strongly recommend all departments consider adopting Daily Contouring Rounds as a means to mitigate risk in the treatment planning process.

## AUTHORs’ CONTRIBUTION

**Adam C. Riegel, PhD**: Participated in all aspects of the study. The author has granted final approval of the version to be published and agrees to be accountable for all aspects of the work in ensuring that questions related to the accuracy or integrity of any part of the work are appropriately investigated and resolved.

**Cynthia Polvorosa, MS**: Participated in methodology, analysis, drafting of manuscript. The author has granted Final approval of the version to be published and agrees to be accountable for all aspects of the work in ensuring that questions related to the accuracy or integrity of any part of the work are appropriately investigated and resolved.

**Anurag Sharma, MS**: Participated in methodology, analysis, drafting of manuscript. The author has granted final approval of the version to be published and agrees to be accountable for all aspects of the work in ensuring that questions related to the accuracy or integrity of any part of the work are appropriately investigated and resolved.

**Jameson Baker, PhD**: Participated in methodology, analysis, drafting of manuscript. The author has granted final approval of the version to be published and agrees to be accountable for all aspects of the work in ensuring that questions related to the accuracy or integrity of any part of the work are appropriately investigated and resolved.

**William Ge, MS**: Participated in methodology, analysis, drafting of manuscript. The author has granted final approval of the version to be published and agrees to be accountable for all aspects of the work in ensuring that questions related to the accuracy or integrity of any part of the work are appropriately investigated and resolved.

**Joseph Lauritano, MS**: Participated in methodology, analysis, drafting of manuscript. The author has granted final approval of the version to be published and agrees to be accountable for all aspects of the work in ensuring that questions related to the accuracy or integrity of any part of the work are appropriately investigated and resolved.

**Emel Calugaru, MS**: Participated in methodology, analysis, drafting of manuscript. The author has granted final approval of the version to be published and agrees to be accountable for all aspects of the work in ensuring that questions related to the accuracy or integrity of any part of the work are appropriately investigated and resolved.

**Jenghwa Chang, PhD**: Participated in methodology, analysis, drafting of manuscript. The author has granted final approval of the version to be published and agrees to be accountable for all aspects of the work in ensuring that questions related to the accuracy or integrity of any part of the work are appropriately investigated and resolved.

**Jeffrey Antone, CMD**: Participated in methodology, analysis, drafting of manuscript. The author has granted final approval of the version to be published and agrees to be accountable for all aspects of the work in ensuring that questions related to the accuracy or integrity of any part of the work are appropriately investigated and resolved.

**Angela Oliviera**: Participated in methodology, analysis, drafting of manuscript. The author has granted Final approval of the version to be published and agrees to be accountable for all aspects of the work in ensuring that questions related to the accuracy or integrity of any part of the work are appropriately investigated and resolved.

**Walkiria Buckenberger**: Participated in methodology, analysis, drafting of manuscript. The author has granted final approval of the version to be published and agrees to be accountable for all aspects of the work in ensuring that questions related to the accuracy or integrity of any part of the work are appropriately investigated and resolved.

**William Chen, MD**: Participated in conception and design of the work. The author has granted final approval of the version to be published and agrees to be accountable for all aspects of the work in ensuring that questions related to the accuracy or integrity of any part of the work are appropriately investigated and resolved.

**Yijian Cao, PhD**: Participated in conception and design of the work. The author has granted final approval of the version to be published and agrees to be accountable for all aspects of the work in ensuring that questions related to the accuracy or integrity of any part of the work are appropriately investigated and resolved.

**Ajay Kapur, PhD**: Participated in conception and design of the work. The author has granted final approval of the version to be published and agrees to be accountable for all aspects of the work in ensuring that questions related to the accuracy or integrity of any part of the work are appropriately investigated and resolved.

**Louis Potters, MD**: Participated in conception and design of the work. The author has granted final approval of the version to be published and agrees to be accountable for all aspects of the work in ensuring that questions related to the accuracy or integrity of any part of the work are appropriately investigated and resolved.

## CONFLICT OF INTEREST

The authors declare that there is no conflict of interest that could be perceived as prejudicing the impartiality of the research reported.
